# KannadaLit4NLP: A comprehensive classical kannada literary dataset of Vachanas, Tripadis, and Kagga with scholarly interpretations for natural language processing

**DOI:** 10.1016/j.dib.2026.112983

**Published:** 2026-06-19

**Authors:** Basavanna C, S. Manjunath, D.S. Guru

**Affiliations:** a Department of Studies in Computer Science, Manasagangotri, University of Mysore, Mysuru 570006, Karnataka, India; b Samsung Electro Mechanics Co Ltd, Bengaluru 560055, Karnataka, India

**Keywords:** Kannada, Low-resource language, Literary corpus, Parallel text dataset, Semantic interpretation, Cultural dataset, Digital humanities

## Abstract

This article presents KannadaLit4NLP, a large-scale, machine-readable corpus of Kannada literary texts designed to support natural language processing (NLP) research for a low-resource language. The dataset comprises 24,746 literary verses from three major Kannada literary traditions—Vachanas (11th–19th century), Tripadis (16th century), and Kagga (20th century)—along with 22,369 corresponding interpretations curated from scholarly sources. The corpus captures linguistic, stylistic, and semantic variations across historical periods and literary forms.

The dataset was developed through a systematic pipeline that included source identification, digitisation via optical character recognition (OCR), manual verification, and structured annotation. Each entry is organised in a structured format that includes the original verse, metadata (literary form, author, and source), and associated interpretation(s), enabling its use in tasks such as semantic textual similarity, textual entailment, information retrieval, and generative modelling.

KannadaLit4NLP addresses the limited availability of culturally grounded Kannada datasets by providing a resource that integrates classical and modern literary content with interpretative annotations. The dataset can facilitate the development and evaluation of NLP models in areas such as semantic understanding, translation, and knowledge representation, while also supporting computational studies of literary and cultural texts. The dataset is made publicly available to encourage further research and reproducibility in Kannada NLP.

Specifications Table

**Table:** 

Subject	Computer Sciences
Specific subject area	Classical Kannada literary corpus for semantic NLP and generative AI
Type of data	Textual data - JSONL (.jsonl) format
Data collection	**Vachana texts:** Extracted from the Sanchaya digital repository and corrected manually against the Samagra Vachana Samputa (Vols. 1–15), Kannada Pustaka Pradhikara, Government of Karnataka (2001). **Tripadi texts:** Digitised via OCR from Sarvajñana Vachanagalu (Ed. Channappa Uttangi, Kannada Sahitya Parishattu, 2019) and verified against the printed edition. **Kagga texts:** Sourced from the Kanaja website (https://kanaja.karnataka.gov.in/kagga/) and cross-verified with the printed edition (Ed. Kavithakrishna, Tanu Manu Prakashana, 2016). **Interpretations:** Digitised from 56 published scholarly volumes (Vachanas and Tripadis) and from the Kanaja website and a dedicated mobile application (Kagga). Image capture was performed using a Samsung Galaxy M31 smartphone (64 MP). OCR used i2OCR and Google Translate (Android). All outputs were manually verified against source editions.
Data source location	**Primary text sources:** • Samagra Vachana Samputa (Vols. 1–15), Kannada Pustaka Pradhikara, Government of Karnataka (2001); • Sarvajñana Vachanagalu, Kannada Sahitya Parishattu (2019); • Mankutimmana Kagga, Tanu Manu Prakashana (2016). **Digital repositories consulted:** • Sanchaya (https://vachana.sanchaya.net/); • Kanaja, Dept. of Kannada and Culture, Govt. of Karnataka (https://kanaja.karnataka.gov.in/kagga/). • Interpretations sourced from 56 published scholarly volumes obtained from libraries, bookshops, Basava Adhyayana Peetha, and Maths/Temples across Karnataka.
Data accessibility	Repository name: Mendeley data - KannadaLit4NLP: A Large-Scale Kannada Literary Corpus for Natural Language Processing Data identification number: 10.17632/nvjydxpxjr.3 Direct URL to data: https://data.mendeley.com/datasets/nvjydxpxjr/3 Users can download the complete dataset from the above repository. The archive is organised into two components. The primary folder, full_dataset, contains 24,746 individual JSONL files (id_1.jsonl to id_24,746.jsonl), one per literary verse, each storing the verse metadata and all available scholarly interpretation–source pairs in a uniform, machine-readable format. For bulk processing, a consolidated master file, KannadaLit4NLP_master.jsonl, is also provided at the root level, containing all 24,746 records as a single JSONL file with one JSON object per line. A README.md documentation file is included to provide field descriptions, corpus statistics, and sample Python scripts for loading and processing the dataset. In addition, a SOURCE.md file provides the complete bibliographic record of all primary literary texts, digital repositories, and interpretive sources used to construct the dataset.
Related research article	1. C. Basavanna, S. Manjunath, D.S. Guru, Retrieval of Kannada Vachanas Based on Philosophical Meaning Using Machine Learning, Data Anal. Learn*.* 1540 (2024) 221–234. https://doi.org/10.1007/978–981–96–9533–1_16 2. C. Basavanna, S. Manjunath, D.S. Guru, Mapping Interpretations to Vachanas: A Text Classification based Approach, Int. Conf. Intell. Comput. Commun. Syst. (2025) 1–6. https://doi.org/10.1109/CICCS66437.2025.11280197

## 1 Value of the Data

• **Unified literary corpus:** To the best of our knowledge, KannadaLit4NLP provides the first unified large-scale digital corpus combining three major Kannada literary traditions—Vachanas, Tripadis, and Kagga—within a consistently structured machine-readable format.
• **Broad NLP utility:** The dataset supports a wide range of Kannada natural language processing tasks, including semantic retrieval, text similarity modelling, meaning-preserving machine translation, question answering, and generative language modelling.
• **Cross-register learning:** The explicit alignment between classical Kannada verses and their modern scholarly interpretations enables computational models to bridge stylistic and linguistic gaps between metaphor-rich literary expressions and explanatory contemporary prose.
• **Digital humanities research:** The dataset facilitates thematic analysis, lexical studies, authorship-based comparison, and examination of linguistic evolution across multiple centuries of Kannada literary history.
• **Generative interpretation testbed:** The presence of 15,149 verses without associated interpretations provides a valuable resource for automatic interpretation generation, explanation synthesis, and evaluation of generative AI systems on culturally grounded literary text.

## 2 Background

Despite recent advances in multilingual NLP, Kannada language remains a relatively under-resourced language in terms of publicly available datasets, benchmarks, and language technologies. Spoken by > 50.8 million people [1] and recognised as one of India's eight classical languages, Kannada possesses a literary tradition spanning >15 centuries [2]. However, most existing computational resources are derived from web content, news articles, Wikipedia text, and parallel translation corpora [[3], [4], [5]]. Kannada is absent from major retrieval benchmarks such as BEIR [6], MIRACL [7], and Mr. TyDi [8]. While AI4Bharat IndicNLP [9] and IndicGLUE [4] have made important contributions, their coverage of Kannada is predominantly modern and does not adequately represent the linguistic, stylistic, and cultural characteristics of classical literary texts.

Classical Kannada literature poses challenges that differ substantially from those in contemporary language processing. Literary forms such as Vachanas, Sarvajna’s Tripadis, and Kagga employ metaphorical expression, philosophical reasoning, symbolic imagery, and culturally grounded references that often extend beyond the text’s literal meaning. Consequently, understanding these works often requires access to scholarly interpretations that explain their ethical, spiritual, social, and philosophical significance. The relationship between a verse and its interpretation, therefore, offers a valuable resource for computational studies of semantic understanding, explanation generation, information retrieval, textual similarity, and knowledge representation.

The three literary traditions in this dataset were selected for their representation of major stages in the evolution of Kannada literary thought and expression. Vachanas, composed between the 11th and 19th centuries by numerous Śaraṇas and saints, constitute one of the largest bodies of Kannada devotional and reformist literature. Sarvajna’s Tripadis represent a distinct tradition of concise wisdom literature expressed in three-line poems. Mankutimmana Kagga, commonly known as Kagga, is a twentieth-century literary work by D. V. Gundappa that offers a modern philosophical perspective while retaining strong ties to classical Kannada thought. Together, these traditions capture linguistic variation across historical periods, literary genres, and philosophical schools, providing a diverse foundation for computational analysis.

To facilitate research in this area, we present KannadaLit4NLP, a large-scale, machine-readable corpus comprising 24,746 literary verses and 22,369 scholarly interpretations, collected from authoritative printed and digital sources. The dataset combines original literary texts, descriptive metadata, and linked interpretations in a unified, structured format designed for computational processing. By integrating classical literary content with scholarly explanatory material, KannadaLit4NLP provides a resource for both NLP and digital humanities research, while contributing to the broader development of language technologies for Kannada and other low-resource languages.

Although several publicly available resources have contributed to the development of Kannada language technologies, they primarily focus on contemporary text, lexical resources, benchmark tasks, or multilingual translation corpora. In contrast, resources that combine classical Kannada literary texts with scholarly interpretative content remain limited. To contextualise the contribution of KannadaLit4NLP within the existing Kannada NLP ecosystem, Table 1 compares the proposed dataset with representative publicly available Kannada language resources and multilingual benchmarks relevant to NLP research.

**Table 1:** Comparison of KannadaLit4NLP with existing publicly available Kannada language resources.

Dataset / Resource	Primary Domain	Kannada Support	Literary Texts	Scholarly Interpretations	Publicly Available	Potential NLP Applications
IndicNLP Corpus [9]	General web and monolingual text	Yes	No	No	Yes	Language modelling, embeddings, text classification
IndicGLUE [4]	NLP benchmark tasks	Yes	No	No	Yes	Classification, NLI, sentence representation
Samanantar [5]	Parallel translation corpus	Yes	No	No	Yes	Machine translation
KannadaLex [1]	Lexical resource with psycholinguistic information	Yes	Limited	No	Yes	Lexical analysis, psycholinguistic studies
BEIR [6]	Information retrieval benchmark	No Kannada dataset	No	No	Yes	Retrieval evaluation
Mr. TyDi [8]	Multilingual retrieval benchmark	No Kannada dataset	No	No	Yes	Dense retrieval evaluation
MIRACL [7]	Multilingual retrieval benchmark	No Kannada dataset	No	No	Yes	Information retrieval
**KannadaLit4NLP**	Classical Kannada literary corpus	Yes	**Yes**	**Yes**	**Yes**	Semantic retrieval, interpretation generation, textual similarity, question answering, generative AI, digital humanities

Beyond the feature-level distinctions shown in Table 1, KannadaLit4NLP differs from existing resources in a fundamental architectural respect. Corpora such as IndicNLP and Samanantar are derived primarily from contemporary text sources, including web content, news articles, and modern parallel translations, and therefore operate within a single linguistic register. In contrast, KannadaLit4NLP is structured as a parallel corpus linking classical Kannada literary verses with modern scholarly interpretations. This verse–interpretation alignment creates a naturally occurring cross-register parallel resource in which thematically linked content is expressed through substantially different linguistic forms. Consequently, KannadaLit4NLP supports Cross-Register Domain Adaptation, enabling computational models to bridge the lexical, stylistic, and syntactic differences between historical literary Kannada and contemporary prose. Such a capability is not available in contemporary-only corpora, regardless of their scale. While existing resources primarily support modern-language tasks such as language modelling, text classification, and machine translation, KannadaLit4NLP also enables research into classical-to-modern semantic alignment, interpretation generation, retrieval-augmented literary analysis, and diachronic studies of Kannada language usage spanning nine centuries of literary production. This cross-register parallel structure constitutes the core architectural differentiator of KannadaLit4NLP and extends the scope of Kannada NLP resources beyond contemporary-language corpora.

## 3 Data Description

The dataset comprises 24,746 individual JSONL files, each representing a single literary verse along with its associated interpretation(s). A single-file-per-verse design was adopted to facilitate verse-level retrieval, simplify version control, support incremental corpus expansion, and enable downstream NLP workflows that operate on individual documents. This organisation also allows researchers to efficiently access, modify, or extend specific records without requiring the processing of the entire corpus. Files are named sequentially as id_N.jsonl, where N corresponds to the id field of the record (e.g., id_1. jsonl, id_194.jsonl, id_2458.jsonl).

To support large-scale processing and improve accessibility, two bulk access options are provided. A consolidated master file, KannadaLit4NLP_master.jsonl, is included at the root of the repository, containing all 24,746 records as a single JSONL file with one JSON object per line. This format supports memory-efficient line-by-line streaming and is directly compatible with standard machine learning tools, including pandas, HuggingFace Datasets, and PyTorch DataLoader. In addition, the complete corpus is available as a single compressed archive (KannadaLit4NLP.zip) in the Mendeley Data repository for one-step bulk download. Both formats are suited for corpus-level experiments, batch inference, and large-scale NLP model training.

The corpus includes 21,701 Vachanas, 2099 Tripadis, and 946 Kagga verses, collectively spanning classical to modern Kannada literature. A total of 22,369 interpretations is linked to 9597 verses, while 15,149 verses (61.2%) currently lack interpretations, reflecting the uneven coverage of scholarly commentary across the corpus, as shown in Table 2.

**Table 2:** Interpretation coverage across literary categories.

Literary Category	Total Verses	Verses with Interpretations	Verses without Interpretations	Coverage (%)
Vachanas	21,701	6833	14,868	31.49
Tripadis	2099	1819	280	86.66
Kagga	946	945	1	99.89
Total	24,746	9597	15,149	38.78

Interpretations were linked to verses through a manual alignment process using verse numbers, author information, and source references as primary matching criteria. Where interpretation sources followed alternative numbering conventions or editorial arrangements, the alignment was verified manually against the original published editions. Multiple interpretations of the same verse were preserved independently and retained without modification, thereby reflecting the diversity of scholarly perspectives in the source literature.

Each JSONL file follows the standard JSON Lines format, where each line contains a single JSON object corresponding to one verse. When multiple interpretations are available, they are stored as sequential key–value pairs (e.g., interpretation1, source1, interpretation2, source2) within the same JSON object. This flat structure ensures simplicity, consistency, and ease of parsing across programming environments. The repository is organised as follows:

**KannadaLit4NLP/**
├—— full_dataset/
│ ├—— id_1.jsonl
│ ├—— id_2.jsonl
│ ├—— …
│ └—— id_24,746.jsonl
└—— KannadaLit4NLP_master.jsonl
└—— README.md
└—— SOURCE.md

The full_dataset folder contains all 24,746 individual JSONL files. The KannadaLit4NLP_master.jsonl file at the root provides the complete corpus in a single file for bulk processing. The README.md file includes field descriptions, corpus statistics, usage notes, and sample Python code for loading and parsing the dataset in multiple formats, including plain Python, pandas, and HuggingFace Datasets. Source references for all verses and interpretations are listed in the SOURCE.md file.

Each JSON object contains the core fields described in Table 3. The interpretation–source pairs follow a flexible schema using pattern-matched keys (interpretationN, sourceN), allowing an arbitrary number of paired annotations per record without requiring a fixed column count.

**Table 3:** Fields contained in each JSON object in the KannadaLit4NLP JSONL files.

The schema below formally describes the flexible, extensible structure used in the dataset, allowing an arbitrary number of interpretation–source pairs per record:

{
"type": "object",
"required": ["id", "volume number", "verse number", "author", "type", "verse"],
"properties": {
"id": {
"type": "integer",
"description": "Unique identifier for the record"
},
"volume number": {
"type": ["integer", "null"],
"description": "Volume number of the source text"
},
"verse number": {
"type": "integer",
"description": "Original verse number"
},
"author": {
"type": "string",
"description": "Author of the verse"
},
"type": {
"type": "string",
"enum": [""],
"description": "Literary category"
},
"verse": {
"type": "string",
"description": "Original Kannada verse text"
}
},
"patternProperties": {
"^interpretation[0-9]+$": {
"type": "string",
"description": "Scholarly interpretation"
},
"^source[0-9]+$": {
"type": "string",
"description": "Source of the interpretation"
}
},
"additionalProperties": false
}

A representative excerpt of the dataset is presented in Table 4. Each row contains a literary verse and its associated metadata, including a unique identifier (id), volume number, verse number, author, and literary category (type). When available, one or more scholarly interpretations are linked to the verse via interpretation–source pairs, illustrating the structure used to represent interpretative information within the corpus.

**Table 4:** A part of the dataset as an example.

Table 5 summarises the scale and linguistic characteristics of each sub-corpus. The full dataset contains 2414,716 tokens across a vocabulary of 466,356 unique types, with the interpretation layer (1627,143 tokens) substantially larger than the verse layer (787,573 tokens), reflecting the discursive nature of scholarly commentary relative to the condensed literary originals.

**Table 5:** Scale and linguistic characteristics of KannadaLit4NLP by sub-corpus.

Category	Time Period	Volume	No. of Authors	Tokens	Vocabulary Size	Avg. Tokens per Verse /Poem/ Interpretation	Characteristics
Vachanas	11th–19th century	21,701	248	752,404	220,135	∼35	Dialogic, spiritual, reformist, metaphor-rich
Sarvajna’s Tripadis	16th century	2099	1	22,650	12,415	∼11	Concise wisdom literature in 3-line form
Kagga	20th century	946	1	12,519	9255	∼13	Ethical, philosophical, modern poetic reflections in 4-line form
Vachanas + Tripadis + Kagga	-	24,746	250	787,573	235,007	-	-
Interpretations	-	22,369	Multiple scholars	1627,143	282,148	∼73	Semantic explanations
Vachanas + Tripadis + Kagga + Interpretations	-	-	-	2414,716	466,356	-	-

The distribution of interpretations across verses is highly skewed, reflecting uneven scholarly attention. Table 6 details this distribution. Out of 24,746 verses, 15,149 (61.2%) have no published interpretation; the remaining 9597 have between 1 and 15 interpretations, with highly canonical verses attracting the most commentary.

**Table 6:** Distribution of scholarly interpretations per verse across KannadaLit4NLP.

Interpretations per verse	No. of verses	Total interpretations
0	15,149	0
1	4439	4439
2	2236	4472
3	767	2301
4	1021	4084
5	535	2675
6	248	1488
7	147	1029
8	84	672
9	51	459
10	31	310
11	21	231
12	14	168
13	2	26
15	1	15
**TOTAL**	**24,746**	**22,369**

**Fig. 1:**
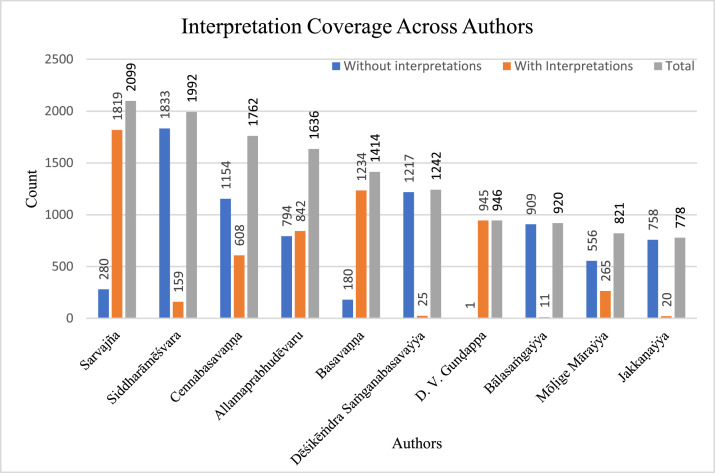
Interpretation coverage across the top 10 authors in the KannadaLit4NLP corpus, showing the number of verses with and without available interpretations, along with the total verses per author.

Fig. 1 presents interpretation coverage for the top 10 authors in the corpus by the number of verses authored, showing for each author the total number of verses written, the number with at least one scholarly interpretation, and the number remaining uninterpreted. While several major authors have a substantial proportion of interpreted verses, coverage varies considerably across authors, with many remaining largely uninterpreted, consistent with the overall skew observed in Table 6.

## 4 Experimental Design, Materials and Methods

Dataset construction proceeded through four phases over nearly six years.

• **Phase 1 — Source Identification and Acquisition.**

Sources were selected on the basis of scholarly authority and literary significance. For Vachanas, the Samagra Vachana Samputa (Volumes 1–15; Kannada Pustaka Pradhikara, Government of Karnataka, 2001) was chosen as the primary source. This anthology was compiled following the foundational recovery work of Halakatti, who retrieved texts from manuscripts, palm-leaf records, and oral traditions, as well as the subsequent curatorial and verification work of Kalburgi and collaborators [10]. For Tripadis, the source was Sarvajñana Vachanagalu (ed. Channappa Uttangi, Kannada Sahitya Parishattu, 2019) [11], collected from manuscripts and oral traditions. For Kagga, the Kanaja website (Department of Kannada and Culture, Government of Karnataka) provided the digital base, with verse accuracy and sequencing cross-verified against the printed critical edition (ed. Kavithakrishna, Tanu Manu Prakashana, 2016) [12].

The sources were selected based on scholarly acceptance, editorial reliability, completeness of coverage, and availability for verification. Where multiple editions existed, preference was given to critical or widely recognised editions frequently cited in Kannada literary scholarship. This strategy was adopted to maximise textual authenticity and ensure consistency across the corpus.

• **Phase 2 — Digitisation of Verses.**

Digitisation used intentionally low-cost tools to demonstrate replicability. Printed pages were photographed using a Samsung Galaxy M31 smartphone (64 MP primary camera, f/1.8, PDAF). Two freely available OCR tools converted the images to text: i2OCR (https://www.i2ocr.com/), a web-based platform that supports Kannada script, and Google Translate (Android).

**Fig. 2:**
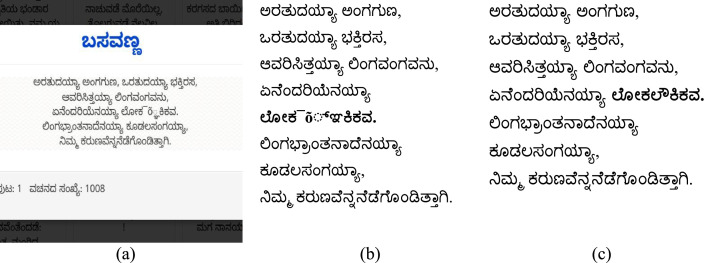
(a). Screenshot of a Vachana sourced from the Sanchaya digital repository [13], showing errors present in the existing digital source. 2(b). Textual rendering of the Vachana from Sanchaya with errors highlighted in bold, showing deviations from the printed Samagra Vachana Samputa. 2(c). Corrected version of the Vachana after manual verification and correction against the printed Samagra Vachana Samputa, showing the final text included in the dataset.

Source-specific approaches were required for dataset construction. For Vachanas, the initial digitised corpus was sourced from the Sanchaya repository [13]. However, comparison with the printed Samagra Vachana Samputa revealed missing verses, transcription errors, and textual inconsistencies, necessitating extensive manual verification and correction. Figs. 2(a) and 2(b) illustrate such discrepancies: Fig. 2(a) shows the digitised version from the Sanchaya repository, while Fig. 2(b) presents the same text with the identified errors highlighted in bold for clarity. To ensure textual accuracy and dataset reliability, all Vachanas were manually cross-checked against the printed Samagra Vachana Samputa, and any detected errors were corrected. The resulting corrected version is shown in Fig. 2(c).

For Tripadis, no reliable digital source existed; the complete text was therefore digitised via OCR from the printed Sarvajñana Vachanagalu. Following OCR digitisation, all extracted text was manually compared against the corresponding source pages. Particular attention was given to Kannada matras, conjunct consonants (ottakṣaras), punctuation marks, verse boundaries, and line segmentation, which were found to be the most common sources of OCR errors. Corrections were made manually by referring to the published work, ensuring that the final text faithfully matches the original publication. For Kagga, the Kanaja website provided the digital base; verse accuracy and sequencing were cross-verified against the printed reference edition.

• **Phase 3 — Collection of Scholarly Interpretations and Digitisation.**

Because the philosophical and ethical meanings of these verses frequently diverge from their literal readings, interpretations were gathered from multiple independent sources. For Vachanas and Tripadis, 56 published books by renowned Kannada scholars were obtained through visits to libraries, bookshops, Basava Adhyayana Peetha, and Maths/ Temples across Karnataka, spanning the state from the southern to the northern regions. For Kagga, interpretations were obtained from a dedicated mobile application and the Kanaja website. All printed interpretation texts were digitised using the same OCR pipeline as Phase 2, with each output manually cross-checked and corrected against the original printed pages.

OCR quality was assessed on random samples of 150 interpretation passages before manual cleaning. The mean Character Error Rate (CER) was 1.33% (range: 0.00%–7.29%), and the mean Word Error Rate (WER) was 13.32% (range: 0.00%–37.93%), when evaluated using the jiwer Python library (v4.0.0). The low CER confirms that individual Kannada character recognition was largely accurate, while the higher WER reflects word-level degradation caused by stray symbols, broken tokens, and misrecognised characters introduced during scanning, necessitating a rigorous manual verification phase against authoritative printed editions.

Interpretations were aligned with verses through a manual verification process primarily based on verse number, author information, and source references. When interpretation sources used alternative numbering systems or editorial arrangements, the corresponding verse was identified and verified manually against the original printed editions. Multiple interpretations associated with a verse were preserved separately and recorded as distinct interpretation–source pairs within the same record. No attempt was made to reconcile, merge, or modify differing scholarly viewpoints. This decision was deliberate, as any alteration to a scholar's interpretive language would compromise the authenticity and scholarly integrity of the corpus. Since interpretations were collected from 56 independent scholarly sources, it is natural that different scholars may interpret the same verse differently. Further, the interpretations are based on philosophical viewpoints of an individual related to social, religious, economical, ethical, political, etc., multidimensional aspects of human life and therefore each verse is having multiple interpretations in different dimensions. The different dimensional interpretations of a verse sometimes seem to be conflicting, but not so in reality, as they are more subjective rather than being objective. Instead of adjudicating between conflicting interpretations or selecting a single authoritative version, we retained all available interpretations as separate named fields within each record (e.g., interpretation_1, source_1, interpretation_2, source_2, and so on, up to 15 pairs per record). This design choice preserves the full diversity of scholarly perspectives and allows downstream researchers to study interpretive variation as a research problem in its own right. Since interpretations were preserved verbatim, stylistic standardisation/normalisation is not generally recommended. Stylistic variation across scholars and publication periods is intentionally retained in the dataset, as it reflects the natural linguistic and scholarly diversity of the Kannada literary commentary tradition.

Scholarly interpretations were mapped to their corresponding verses only after the proofreading phase was fully completed across all entries. This sequential workflow ensured that no interpretation was ever linked to an incorrectly digitised verse. During the redundancy control phase, a total of 1009 interpretation entries were excluded across 56 source books, comprising duplicate interpretations already present from other sources, interpretations whose corresponding verse was not found in the source verse reference editions [[10], [11], [12]], and corrupted or misattributed verse entries. All retained interpretations were preserved exactly as published in the source material. The only modifications applied during dataset construction were correcting OCR transcription errors and applying Unicode normalisation to ensure a consistent digital representation of the Kannada script. No summarisation, semantic editing, simplification, or harmonisation of scholarly interpretations was performed.

• **Phase 4 — Quality Assurance and Normalisation.**

Quality assurance was carried out through multiple rounds of manual verification. Each digitised verse and its interpretation were compared with their source editions to identify OCR-related errors, omissions, formatting inconsistencies, and metadata mismatches. Validation included verification of verse numbering, author attribution, literary category, source references, and interpretation linkage. All texts were Unicode-normalised (NFC form) to ensure consistent encoding of Kannada characters across sources. Table 7 summarises the validation activities conducted to ensure the dataset's correctness.

**Table 7:** Quality assurance activities.

Validation Activity	Description
OCR verification	Comparison against source editions
Metadata validation	Verification of author, verse number, volume
Interpretation alignment	Verification of verse–interpretation mapping
Duplicate detection	Identification and removal of duplicate records
Unicode normalization	NFC standardization
Repository validation	JSONL structure verification

### 4.1 Baseline evaluation of dataset utility

To demonstrate the utility of KannadaLit4NLP for downstream natural language processing applications, an illustrative baseline experiment was conducted. This experiment was not intended to establish state-of-the-art performance, but rather to provide reference baselines and highlight the challenges associated with classical Kannada literary texts and scholarly interpretations. The selected task represents the semantic retrieval application area that the dataset supports. However, more baseline experimentation related details shall be referred to in [2] and [14].

### 4.2 Semantic retrieval of vachanas

One potential application of KannadaLit4NLP is the semantic retrieval of literary verses from natural language descriptions provided by users. This capability can assist scholars, researchers, writers, and public speakers in locating relevant Vachanas, Tripadis, and Kagga without extensive manual search or expert knowledge of classical Kannada literature. The task is particularly challenging because user queries are typically expressed in contemporary Kannada, whereas the corpus comprises classical literary texts and scholarly interpretations.

To establish baseline performance, two retrieval approaches were evaluated. The first approach used a traditional TF–IDF vector space model. Interpretations were pre-processed through tokenisation and text normalisation, then represented as TF–IDF vectors. Retrieval was performed using cosine similarity between the query vector and stored interpretation vectors. The second approach used sentence embeddings generated with KannadaSBERT-STS (l3cube-pune/kannada-sentence-similarity-sbert) [15], a transformer-based model trained for semantic textual similarity in Kannada. Query and interpretation embeddings were compared using cosine similarity, and the top-K most relevant results were retrieved. Upon retrieval, the corresponding Vachanas, Tripadis, or Kagga verses associated with each matched interpretation were displayed to the user as the final output.

For evaluation, 5158 verses with at least two independent interpretations were selected, yielding 17,930 interpretation records. The dataset was split into equal training and test partitions using the train_test_split() function from the Python scikit-learn library, with train_size=0.5 and random_state=42, resulting in 8965 instances in each partition. Stratification by verse identifier (id) was applied to preserve the distribution of interpretations per verse across both subsets. This was feasible because the preceding filtering step ensured that every verse had at least two interpretations. Retrieval performance was assessed using exact-match accuracy, in which a retrieval was considered successful only if the correct target verse appeared among the top-K retrieved results [2]. K was varied from 1 to 20 to evaluate retrieval effectiveness at different depths.

**Fig. 3:**
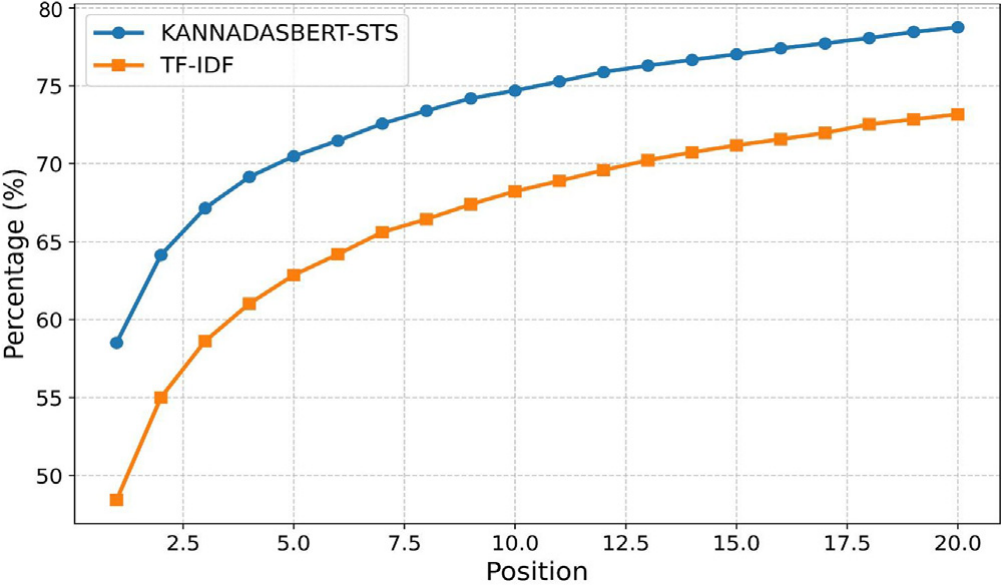
Exact-match retrieval performance of TF-IDF and KannadaSBERT-STS across Top-1 to Top-20 retrieval ranks.

The retrieval results are presented in Fig. 3. The TF–IDF baseline achieved an exact-match accuracy of 48.36% at Top-1 retrieval, increasing to 73.22% at Top-20. The KannadaSBERT-STS embedding approach consistently outperformed the TF–IDF baseline, achieving 58.10% at Top-1, 75.00% at Top-10, and 78.41% at Top-20 retrieval. The superior performance of the embedding-based approach suggests that semantic representations are more effective in capturing the contextual and conceptual relationships between contemporary Kannada queries and classical literary interpretations. These results demonstrate the potential of KannadaLit4NLP as a benchmark resource for semantic retrieval and literary knowledge discovery in Kannada.

## Limitations

A total of 61.2% of verses lack interpretations, limiting supervised learning applications. OCR errors may persist despite manual correction. Interpretations reflect individual scholarly perspectives and may vary. Because the corpus consists primarily of classical and literary Kannada, models trained exclusively on this dataset may not generalise effectively to contemporary Kannada used in news media, social media platforms, conversational communication, or other modern domain-specific applications without additional domain adaptation. The dataset does not include translations, linguistic annotations, or syntactic and morphological labels.

## Ethics Statement

This dataset does not involve human participants, animal experiments, social media data, or personally identifiable information. The corpus was compiled from published literary works, scholarly commentaries, and publicly accessible digital resources for academic research purposes. All source materials are acknowledged and documented in the accompanying source reference file. It is indeed an attempt to make such scholarly materials available for researchers to experiment and explore towards the generation of new knowledge in support of possessing of the regional language

Kannada by spreading the verses to the computing scientific community. We confirm that we have read and followed the publication guidelines in Data in Brief.

## CRediT Author Statement

**Basavanna C.:** Data collection, Data curation, Digitisation, Verification, Writing – original draft; **S. Manjunath:** Methodology, Writing – review and editing; **D. S. Guru:** Conceptualisation, Methodology, Formal analysis, Supervision, Writing – review and editing.

## Data Availability

Mendeley DataKannadaLit4NLP: A Large-Scale Kannada Literary Corpus for Natural Language Processing (Original data).
